# Development of an administrative definition for celiac disease

**DOI:** 10.1186/s13104-019-4693-2

**Published:** 2019-10-17

**Authors:** Donald R. Duerksen, Lisa M. Lix, William D. Leslie

**Affiliations:** 1 Department of Internal Medicine, Rady Faculty of Health Sciences, University of Manitoba, Winnipeg, MB Canada; 2 Department of Community Health Sciences, Rady Faculty of Health Sciences, University of Manitoba, Winnipeg, MB Canada; 3 Department of Medicine, Division of Gastroenterology, St. Boniface Hospital, C5120 409 Tache Ave, Winnipeg, MB R2H 2A6 Canada

**Keywords:** Celiac disease, Health administrative data, Epidemiology

## Abstract

**Objective:**

The investigation and management of celiac disease places a high burden on the health care system. Accurate methods to ascertain cases of celiac disease (CD) in population-based administrative data can facilitate epidemiologic and health services research to guide disease management. The study aim was to develop and validate administrative data case definitions for CD to facilitate further studies about the effect of CD on osteoporosis and fracture risk.

**Results:**

Population-based data from the Manitoba Bone Mineral Density (BMD) Program registry, which contains medical information on all individuals in the province of Manitoba, Canada who have received BMD testing, was used to define the study cohort. Linked hospital discharge abstracts and physician billing claims were used to ascertain diagnoses of celiac disease in administrative data. A population-based CD serologic registry was used as the validation database. One diagnosis code in hospital discharge abstracts or two or more diagnosis codes in physician billing claims optimized the detection of positive celiac serology with sensitivity of 84% (95% CI 80–88%), specificity of 97% (95% CI 80–88%), PPV of 80% (95% CI 80–88%), and NPV of 97% (95% CI 80–88%). Our administrative data case definition for celiac disease demonstrates good sensitivity and specificity for detecting positive celiac serology.

## Introduction

Celiac disease is a common intestinal disorder affecting an estimated 1% of individuals in North America [1]. This multisystem disease affects multiple organ systems and is associated with an increased prevalence of autoimmune disorders, gastrointestinal malignancies and osteoporosis [2]. Given these factors, there is considerable burden on the health care system. Disease associations and outcomes can be studied using population-based administrative health data where a suitable case definition for the condition of interest has been developed and validated. Case definitions showing high sensitivity and excellent specificity have been developed in other chronic conditions such as inflammatory bowel disease, multiple sclerosis and diabetes mellitus [3–5].

One of the disease associations relevant to celiac disease is osteoporosis and increased fracture risk [6–9]. The development of a case definition of celiac disease would facilitate further study of the effect of celiac disease on bone disease. The aim of this study was to develop case definitions for celiac disease using population-based administrative health data that would allow for investigation of celiac disease and osteoporosis/fracture relationships.

## Main text

### Methods

The study cohort was developed from the Manitoba Bone Mineral Density Database. The population-based Manitoba BMD Program registry contains information on all individuals in the province of Manitoba, Canada who have received BMD testing with dual-energy X-ray absorptiometry (DXA). The accuracy and completeness of the BMD registry exceeds 99% and has been well described [10]. In addition to complete data on BMD and risk factors for fracture, this database has been linked to multiple provincial administrative health databases including the Manitoba Celiac Serology Registry. The study protocol was approved by the University of Manitoba Health Research Ethics Board. Data access was approved by the Health Information Privacy Committee.

Administrative health databases in Manitoba capture virtually all physician claims and all hospitalizations on provincial residents eligible to receive health services. Physician claims are submitted to the provincial ministry of health by physicians paid on a fee-for-service basis; they capture virtually all outpatient services and contain a single diagnosis code recorded using the International Classification of Diseases, 9th revision, Clinical Modification (ICD-9-CM). Only the first three digits of the ICD-9-CM code are recorded in physician billing claims data, which is insufficient to definitely distinguish celiac disease from other causes of intestinal malabsorption. Hospital discharge abstracts are completed at discharge from acute care facilities and contain up to 16 diagnoses coded using ICD-9-CM up to March 31, 2004 and up to 25 diagnoses coded using the Canadian Adaptation of the 10th revision of ICD (i.e., ICD-10-CA) after this date. There is no restriction on the number of digits/characters used to record diagnoses in hospital abstracts; hence, celiac disease can be distinguished from the other causes of intestinal malabsorption in this database.

Physician billing claims and hospital discharge abstracts can be linked via an encrypted personal health number to other administrative data sources. As well, they can be linked to the population registry, which contains information about dates of health insurance coverage, demographic characteristics, and location of residence.

Repository databases were used to identify the number of physician billing claims (P) and hospital discharge abstracts (H) related to celiac disease and malabsorption disorders from the ICD codes noted above. The index date for each case was defined as the date of the BMD test.

#### Celiac disease serologic registry

Central to the diagnosis of celiac disease is serologic testing [11]. While the definitive diagnosis of celiac disease requires a confirmatory small intestinal biopsy in adults, IgA anti tissue transglutaminase (TTG) antibodies have shown a high sensitivity (92.8%) and specificity (97.9%) for the diagnosis of celiac disease [12]. Similarly, the IgA anti-endomysial antibodies (EMA) have a very high specificity for celiac disease (99%) but a lower sensitivity (73%) than TTG antibodies [12–14]. All serologic testing for celiac disease in Manitoba (1.3 million people) is performed by a central immunology lab located in Winnipeg, the largest urban centre in the province. Serologic testing with EMA testing has been performed in this laboratory since 1996. Tissue transglutaminase testing has been available since 2003. A seropositive case was defined as TTG and EMA positive (since 2003) or EMA positive (prior to 2003) on serology performed prior to BMD testing. A seronegative case was defined as being EMA negative. For individuals who had multiple tests the highest positive EMA reading was considered.

#### Administrative case definitions

Case definitions were developed using hospital discharge abstracts and/or physician billing claims (denoted P). Administrative data was examined up to 3 years prior to the index date, the date of the BMD test. For hospitalizations, we examined coding at the less specific integer level (ICD-9-CM 579 or ICD-10-CA K90, denoted H) and coding at the more specific decimal level (ICD-9-CM 579.0 or ICD-10-CA K90.0, denoted H*). Case definitions using different combinations of administrative data contacts were developed. For example, H1P2 denotes 1 hospital discharge abstract (integer level) or 2 physician claims; H1*P3 denotes 1 discharge abstract (decimal level) or 3 physician claims.

#### Statistical analysis

The study cohort was described using frequencies, percentages, means, and standard deviations. Chi square tests of independence and t-tests were used to test for differences between seropositive cases and seronegative non-cases on socio-demographic and comorbidity characteristics. Estimates of sensitivity, specificity, positive predictive value (PPV), negative predictive value (NPV), and overall accuracy for each case definition were computed, along with 95% confidence intervals (95% CIs).

### Results

We conducted sensitivity analyses stratified by age (< 50 years vs > 50 years), sex (female vs male), area of residence (urban vs rural), calendar year (before 2006 vs after 2006), level of comorbidity and income quintile group. Comorbidity was defined using Aggregated Diagnosis Groups™ (ADGs™) codes from the Johns Hopkins Adjusted Clinical Group^®^ (ACG^®^) Case-Mix System version 9. Low comorbidity was denoted by an ADG score <6 whereas high comorbidity was denoted by an ADG score of 6 or greater [15]. Mean household income, based upon area of residence in the year of the DXA test, was extracted from the public use files of the Statistics Canada Census for 2006. Lower income was denoted by household income in the lower two quintiles while higher income was denoted by household income in the upper three quintiles as previously described [16].

There were 250 seropositive cases (14%) and 1588 seronegative non-cases (86%). The median time between dates of serologic and BMD testing was 1.6 years (interquartile range 0.7–2.9 years). Table 1 summarizes socio-demographic and comorbidity characteristics of cases and non-cases. Seropositive cases tended to be younger, have a lower comorbidity score, and a greater percentage were males, compared with seronegative non-cases. There were no statistically significant differences between groups when comparing income quintile group or location of residence. Table 2 reports the sensitivity, specificity, PPV, NPV and accuracy of the different case definitions in predicting positive celiac serology. Diagnosis codes in hospital discharge abstract alone (H1 or H1*) showed low sensitivity [58% (95% CI 50–65) and 56% (95% CI 50–65) respectively] but high PPV (> 80%). Diagnosis codes in physician billing claims showed higher sensitivity but lower specificity, with a reciprocal trade-off for requiring greater numbers of codes (P1 vs P2 vs P3). Utilization of one diagnosis code in hospital discharge abstracts or 2 diagnosis codes physician claims (H1P2) optimized accuracy (95%) for the case definition of celiac disease. Utilization of this definition resulted in high sensitivity (84%) specificity (97%) PPV (80%) and NPV (97%) for positive celiac serology. Results were identical for less specific (H1P2) or more specific coding (H1*P2) in hospital discharge abstracts. The administrative case definition was not influenced by the serologic definition (EMA positive vs EMA/TTG positive). Table 3 compares the performance of the case definition of H1P2 in subgroups defined by age, sex, area of residence, calendar year, level of comorbidity and income quintile group for correctly classifying serologically positive and negative cases.

**Table 1:** Description of the study cohort

	Negative serology	Positive serology	Total	p-value
N	1588	250	1838	
Age, years	59 ± 13.3	46.9 ± 13.7	57.3 ± 13.4	< 0.001
Male	206 (13)	55 (22)	261 (14.2)	< 0.001
Comorbidity index^a^	6.1 ± 2.9	5.2 ± 2.7	6.0 ± 2.9	< 0.001
Rural residence	459 (28.9)	80 (32)	539 (29.3)	0.318
Lower income	538 (33.9)	73 (29.2)	611 (33.2)	0.145

^a^ Number of Aggregated Diagnosis Groups (ADGs). Data are mean ± SD or N (percent)

**Table 2:** Performance of case definitions for celiac seropositivity in administrative health data

	Sensitivity (95% CI)	Positive predictive value (95% CI)	Specificity (95% CI)	Negative predictive value (95% CI)	Accuracy (95% CI)
H1	58 (50–65)	84 (78–89)	98 (96–100)	94 (90–97)	93 (89–97)
H1*	56 (49–64)	84 (79–90)	98 (96–100)	93 (90–97)	93 (89–97)
P1	92 (89–95)	73 (68–78)	95 (92–97)	99 (98–100)	94 (92–97)
P2	78 (73–83)	83 (78–87)	97 (95–99)	97 (94–99)	95 (92–98)
P3	52 (44–59)	84 (78–90)	98 (96–100)	93 (89–97)	92 (88–96)
H1P1	93 (90–96)	71 (66–76)	94 (91–97)	99 (98–100)	94 (91–96)
H1P2	84 (80–88)	80 (75–84)	97 (94–99)	97 (96–99)	95 (92–98)
H1P3	75 (70–81)	82 (77–87)	97 (95–99)	96 (94–99)	94 (91–97)
H1*P1	93 (90–96)	71 (66–76)	94 (91–97)	99 (98–100)	94 (91–96)
H1*P2	84 (80–88)	80 (75–84)	97 (94–99)	97 (96–99)	95 (92–98)
H1*P3	75 (70–81)	82 (77–87)	97 (95–99)	96 (94–99)	94 (91–97)

H, hospitalization diagnosis, number corresponds to frequency of diagnosis codes in hospital discharge abstract that were required to ascertain a positive serology case; P, physician billing claims diagnosis, number corresponds to frequency of diagnosis codes in physician billing claims required to ascertain a positive serology case; H1*, specific code for celiac disease (ICD-9-CM code 679.0, ICD-10-CA code K90.0); CI, confidence interval

**Table 3:** Subgroup performance of the H1P2 case definition

	Sensitivity (95% CI)	Positive predictive value (95% CI)	Specificity (95% CI)	Negative predictive value (95% CI)	Accuracy (95% CI)
All	84% (80–88)	80 (75–84)	97 (94–99)	97 (96–99)	95 (92–98)
Age < 50	90% (86–95)	86 (80–91)	94 (90–97)	96 (93–99)	93 (89–97)
Age > 50	74% (66–83)	70 (61–79)	97 (94–100)	98 (95–101)	96 (92–100)
Female	83% (78–88)	80 (75–86)	97 (95–99)	98 (95–100)	95 (92–98)
Male	87% (79–96)	77 (67–88)	93 (87–99)	96 (92–101)	92 (85–99)
Lower comorbidity	86% (80–91)	84 (78–90)	97 (94–100)	97 (94–100)	95 (91–98)
Higher comorbidity	81% (74–89)	73 (65–82)	96 (93–100)	98 (95–100)	95 (91–99)
Rural	86% (80–91)	86 (79–94)	98 (94–101)	98 (94–101)	96 (92–100)
Urban	83% (78–88)	77 (71–83)	96 (93–99)	97 (95–100)	94 (91–98)
Lower income	86% (79–94)	75 (66–84)	96 (92–100)	98 (95–101)	95 (90–100)
Higher income	83% (78–89)	82 (76–87)	97 (94–99)	97 (95–100)	95 (92–98)
Year < 2006	90% (84–96)	73 (64–81)	94 (90–99)	98 (96–101)	94 (89–99)
Year > 2006	81% (75–87)	84 (78–90)	98 (95–100)	97 (94–100)	95 (92–99)

CI, confidence interval

### Discussion

Clinical registries in Manitoba have been used to validate administrative case definitions for inflammatory bowel disease, diabetes mellitus and multiple sclerosis [3–5]. These definitions form the basis of epidemiologic studies that further evaluate disease associations and outcomes. In the current study we have demonstrated that administrative data related to celiac disease can be used to derive an administrative case definition that shows good agreement with celiac serology.

Establishing a case definition of celiac disease has some inherent challenges. The three-digit ICD-9-CM code present in physician billing claims and used in most provinces in Canada [17] for many jurisdictions is not specific for celiac disease and includes other causes of malabsorption. A previous study examining the role of ICD-9-CM codes in predicting the diagnosis of celiac disease demonstrated a very low PPV when this was used in isolation [18]. More recently, in a pediatric study from Canada, there was excellent specificity (> 99%) but only moderate sensitivity (70.4%) when a claim for a gastroscopy and 1 or more outpatient claims by a pediatric or adult gastroenterologist was made [19]. This was a pediatric study using intestinal biopsy to validate the case definition and included 125 biopsy proven celiac cases. Our study of adult patients demonstrates that using a definition of 1 hospital discharge abstract or 2 outpatient physician claims and increasing the specificity of the ICD-9-CM claims by excluding other less common causes of malabsorption can result in a case definition that is sensitive (84%) and highly specific (97%) in predicting positive celiac serology.

Although our study did not validate the case definition of celiac disease with biopsy positive celiac disease as biopsy data is not part of the database, recent pooled sensitivity and specificity data demonstrate very high sensitivities (92.8%) and specificities (97.9%) of TTG antibodies in predicting biopsy proven celiac disease [12]. EMA antibodies have a lower sensitivity (73%) but a high specificity (99%) for this diagnosis [12]. While there has been variability reported in sensitivities and specificities of celiac serology between laboratories [20], in the province of Manitoba there is one central laboratory that performs all of the testing.

### Conclusion

In conclusion, this study demonstrates that an administrative definition for celiac disease based on ICD correlates well with positive celiac serology. Given the high sensitivity and specificity of celiac serology in predicting celiac disease, this definition could be used in population-based epidemiologic and health services research on this condition.

## Limitations

The current study utilized celiac serology results linked to health claims data in a comprehensive BMD database to establish an administrative definition of celiac disease. There is potential for selection bias as this provincial bone mineral density database included a disproportionate number of elderly, post-menopausal females. This is evident in Table 1 where the celiac negative patients were older, a greater proportion were female and, likely due to their older age, had increased comorbidity. Despite this, performance was robust across multiple subgroups and thus the results should generalize across the population of Manitoba. Further assessment of the generalizability of the results in other Canadian and non-Canadian populations is a future area of research. The other potential source of bias relates to the patients testing negative for celiac disease. While the sample size is relatively large at over 1500 patients, we did not test the generalizability of this population to the non-celiac Manitoba population.

## Data Availability

The datasets used and/or analysed during the current study are available from the corresponding author on reasonable request.

## Abbreviations

BMD: bone mineral density
DXA: dual-energy X-ray absorptiometry
NPV: negative predictive value
PPV: positive predictive value
ICD-9-CM: International Classification of Diseases, 9th revision, Clinical Modification
ICD-10-CA: International Classification of Diseases, 10th revision
P: physician billing claims
H: hospital discharge abstracts
TTG: anti tissue transglutaminase antibody
EMA: anti-endomysial antibodies
ADGs™: Aggregated Diagnosis Groups™
